# The Evolution of Calcification in Reef-Building Corals

**DOI:** 10.1093/molbev/msab103

**Published:** 2021-04-19

**Authors:** Xin Wang, Didier Zoccola, Yi Jin Liew, Eric Tambutte, Guoxin Cui, Denis Allemand, Sylvie Tambutte, Manuel Aranda

**Affiliations:** 1 Biological and Environmental Sciences & Engineering Division (BESE), King Abdullah University of Science and Technology (KAUST), Red Sea Research Center (RSRC), Thuwal, Saudi Arabia; 2 Marine Biology Department, Centre Scientifique de Monaco, Monaco, Monaco

**Keywords:** coral reefs, biomineralization, calcium carbonate skeleton, phylogenomics

## Abstract

Corals build the structural foundation of coral reefs, one of the most diverse and productive ecosystems on our planet. Although the process of coral calcification that allows corals to build these immense structures has been extensively investigated, we still know little about the evolutionary processes that allowed the soft-bodied ancestor of corals to become the ecosystem builders they are today. Using a combination of phylogenomics, proteomics, and immunohistochemistry, we show that scleractinian corals likely acquired the ability to calcify sometime between ∼308 and ∼265 Ma through a combination of lineage-specific gene duplications and the co-option of existing genes to the calcification process. Our results suggest that coral calcification did not require extensive evolutionary changes, but rather few coral-specific gene duplications and a series of small, gradual optimizations of ancestral proteins and their co-option to the calcification process.

## Introduction

Reef-building corals build the largest living structures in the world that provide a habitat for more than a quarter of all marine animals (Fisher et al. 2015), and are the primary source of livelihood to hundreds of millions of people (Spalding et al. 2017). Their immense structures are built through calcification, that is, the continuous deposition of calcium carbonate that forms their skeletons. Calcification is also called biomineralization since aragonite skeletons are biominerals formed both of a mineral fraction consisting of calcium carbonate and a fraction of organic matrix molecules that includes carbohydrates, lipids, and proteins (Falini et al. 2015). Therefore, the calcification process is controlled by the supply of ions required for mineral deposition and the secretion of organic matrix molecules for the organic fraction. It has recently been suggested that the first step of calcification starts intracellularly with the formation of amorphous calcium carbonate (ACC) particles stabilized by organic matrix proteins (Mass et al. 2017; Von Euw et al. 2017; Sun et al. 2020). It has also been shown that pH and the concentrations of calcium and carbonate ions are higher in the extracellular calcifying medium (ECM), where crystals grow, than in seawater (Venn et al. 2019; Sun et al. 2020). Therefore, it has been proposed that membrane transporters and enzymes likely control both the ECM and ionic composition of vesicles by supplying calcium and bicarbonate and by removing protons from these compartments (Sun et al. 2020).

Calcium ions can be transported by several proteins, such as Ca-ATPases, which exchange two calcium ions for four protons across the cell membrane (Ip et al. 1991; Zoccola et al. 1999, 2004). The supply of ${\text{HCO}}_{3}^{-}$ is supported by specific transporters encoded by two distinct membrane protein families, solute carrier 4 (SLC4), and solute carrier 26 (SLC26) (Zoccola et al. 2015). Enzymes belonging to the group of carbonic anhydrases (CAs) can facilitate and catalyze the hydration of metabolic CO_2_ into ${\text{HCO}}_{3}^{-}$ in the ECM (Moya et al. 2008; Zoccola et al. 2015). Organic matrix proteins are secreted by the calicoblastic cells and are supposed to play a role in stabilizing ACC (Von Euw et al. 2017). These proteins can promote nucleation and crystal growth/inhibition of growth, by connecting calicoblastic cells to the skeleton and by allowing species-specific morphological differentiation (Falini et al. 2015). Previous proteomic analyses of coral skeletal organic matrix proteins (SOMPs) by Drake et al. (2013a), Ramos-Silva et al. (2013), and Takeuchi et al. (2016) identified several extracellular matrix-like proteins and domains, including Laminin G, CUB-domain, and EP-like proteins, as well as several transmembrane proteins including cadherin-like, neurexin, EGF domain, zona pellucida domain, and mucin4-like proteins.

Although our understanding of the processes that enable corals to build reefs has significantly improved over the last decades, we still know little about the evolutionary changes that allowed the soft-bodied, anemone-like ancestor of reef-building corals to become the ecosystem builders they are today. Although it has been suggested that their order, Scleractinia, might be as old as >450 Ma (Stolarski et al. 2011; Huang and Roy 2013; Quattrini et al. 2020), the earliest evidence of reef-building corals appears in the fossil record around 265 Ma (Ezaki 2000), with the majority of the fossils clearly identified as Scleractinia dating to the early Triassic ∼240 Ma (Veron 1995; Jin et al. 2000). Analyses of morphological and molecular markers indicate that calcification evolved multiple times independently in the different calcifying cnidarian lineages (Miglietta et al. 2010) and it has been suggested that scleractinian corals might have lost and regained the ability to calcify multiple times during their evolution. A recent study of calcification genes across several hexacorallian transcriptomes, encompassing corals, corallimorphs, and anemones, further suggested that the ability to calcify might not have required extensive genetic adaptations and the evolution of new, specialized proteins (Lin et al. 2017). If this is the case, what is required and how did the noncalcifying ancestor of reef-building corals evolve this ability? To further understand how corals acquired the ability to calcify, we used a comparative genomic approach based on two evolutionary divergent scleractinians (*Acropora digitifera* [Shinzato et al. 2011] and *Stylophora pistillata* [Voolstra et al. 2017]), their closest noncalcifying relatives, the Corallimorpharia (*Amplexidiscus fenestrafer*, *Discosoma* sp. [Wang et al. 2017]), and two sea anemones (*Nematostella vectensis* [Putnam et al. 2007] and *Exaiptasia pallida* [Baumgarten et al. 2015]). We specifically traced the origin and evolutionary history of known calcification genes and protein constituents of the skeletal organic matrix (SOM) to identify the evolutionary innovations that turned the ancestor of corals into the founding species of the iconic coral reef ecosystem.

## Results

### Coral Calcification Evolved Sometime between 308 and 265 Ma

**Fig. 1.**
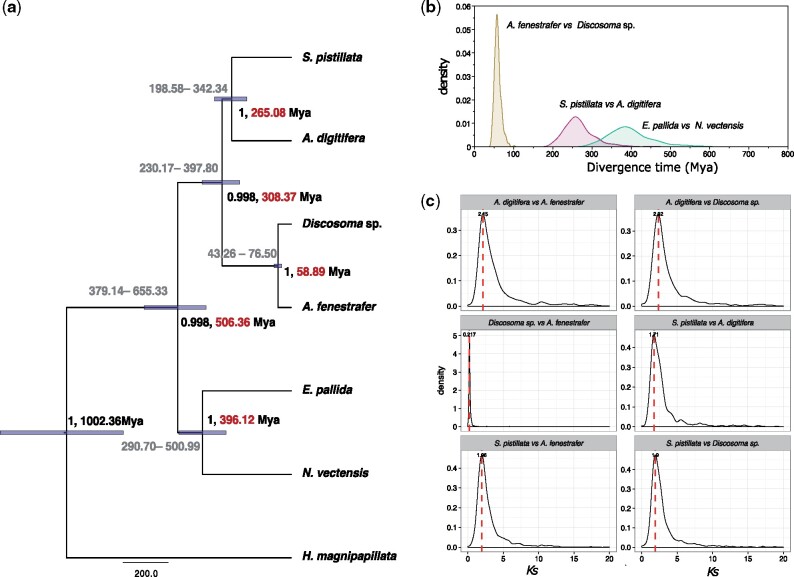
Phylogenetic divergence across seven published cnidarian genomes. (*a*) Phylogenetic analysis of seven cnidarian genomes. The values on nodes indicate the posterior and mean node age. Values on branches show the age range (95% HPD). (*b*) Marginal density of divergence times for three groups, including Scleractinia, Corallimorpharia and Actiniaria. (*c*) Density of pair-wise genetic divergence between two scleractinians and two corallimorpharians calculated based on synonymous substitution rated (*Ks*) across 1,421 orthologous genes.

Based on a phylogenetic analysis using 1,421 single copy orthologs and a time-calibrated tree, our result suggests that Corallimorpharia evolved as a sister group of Scleractinia after their common divergence from Actiniaria approximately ∼506 Ma (±149 Ma, fig. 1*a*). Using the distribution of divergence time estimates based on BEAST and synonymous substitutions per synonymous sites (*Ks*) across orthologs identified in all six hexacorallian genomes, we further estimate that the split between Scleractinia and Corallimorpharia dates back to ∼308 Ma (±78 Ma, fig. 1*a*–*c*). Furthermore, we estimate the divergence of these two coral crown clades to sometime around ∼265 Ma (fig. 1*a* and *b*), which aligns precisely with the appearance of the first Scleractinia-like organisms in the fossil record (Ezaki 2000). Therefore, we conclude that the evolution of scleractinian calcification can likely be placed within a time window of ∼43 Ma between the divergence of Scleractinia from Corallimorpharia ∼308 Ma and the appearance and divergence of the two extant coral clades ∼265 Ma (fig. 1*a*–*c* and supplementary fig. S1, Supplementary Material online).

### The Evolution of Plasma Membrane Calcium ATPase

**Fig. 2.**
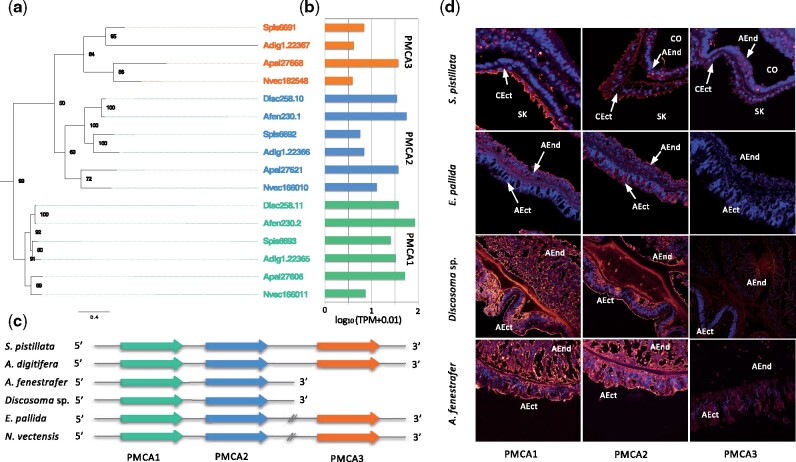
Plasma membrane calcium ATPase (PMCA). (*a*) Phylogeny of PMCA orthologs across hexacorallian genomes. (*b*) Expression of PMCA orthologs across six genomes. (*c*) Synteny of PMCA orthologs. (*d*) Immunolocalization of PMCA1, PMCA2, and PMCA3 in *S. pistillata*, *E. pallida, Discosoma* sp., and *A. fenestrafer*. AEnd, aboral endoderm; AEct, aboral ectoderm; CEct, calicoblastic ectoderm; Co, coelenteron; SK, skeleton.

The transport of calcium to the ECM is thought to be performed by plasma membrane calcium-ATPases, which exchange two calcium ions for four protons across the membrane (Ip et al. 1991; Zoccola et al. 1999, 2004) (supplementary table S1, Supplementary Material online). Searching the six genomes for calcium-transporting ATPases (PMCA), we identified three different genes (PMCA1-3), two of which appear to be hexacorallian-specific gene duplications (fig. 2*a*). Analysis of transcriptomic data further confirmed that these genes are indeed present, or missing, in the respective species (fig. 2*b*). Interestingly, all six genomes encoded at least two of these homologs in tandem (fig. 2*c*). In corals, a third copy (PMCA3) was located adjacently while it was encoded on a different genomic locus in the two anemones and completely absent in the Corallimorpharia genomes (fig. 2*c*). Immunolocalization analyses using cross-hybridizing antibodies showed that PMCA1 and PMCA2 proteins are ubiquitously localized in the Corallimorpharia *A. fenestrafer* and *Discosoma* sp. Similarly, we found PMCA2 also to be ubiquitously localized in the actiniarian *E. pallida* while PMCA1 only showed expression in the aboral endoderm but not the aboral ectoderm in this species. In contrast to this, we found that both PMCA1 and PMCA2 displayed strong localization to the calicoblastic ectoderm in the coral *S. pistillata* (fig. 2*d*). Interestingly, we could not identify PMCA3 expression in *Exaiptasia* despite its clear presence in the genome and transcriptome. However, antibody staining against PMCA3 in the symbiotic sea anemone *Anemonia viridis* did show ubiquitous expression throughout all tissue (supplementary fig. S2, Supplementary Material online), whereas showing localization to small spots of high intensity in the coral *S. pistillata* (fig. 2*d*). It should be further noted that no antibody staining was observed for PMCA3 in Corallimorpharia (fig. 2*d*) in accordance with the absence of this gene in this order (fig. 2*a* and *c*).

### The Evolution of Bicarbonate Transporter

**Fig. 3.**
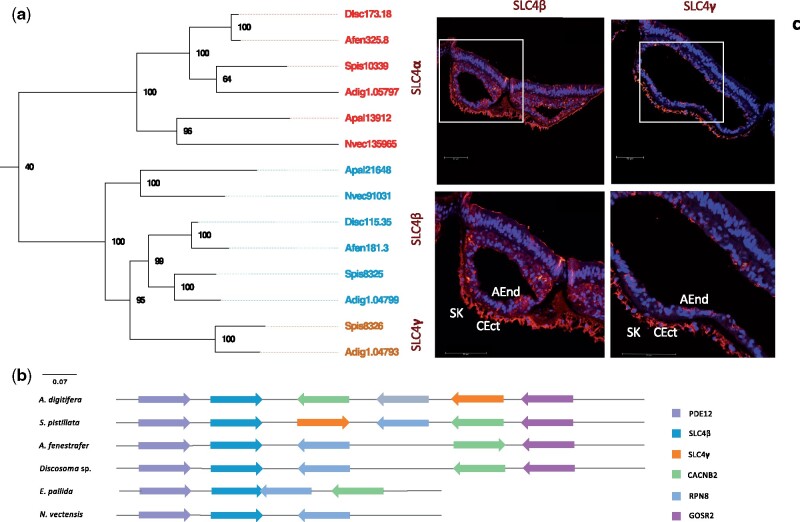
Solute carrier 4. (*a*) Phylogeny of SLC4 across six hexacorallian genomes. (*b*) SLC4β, SLC4γ, and their surrounding genomic locus across six hexacorallian genomes. (*c*) Immunolocalization of SLC4β (left) and SLC4γ (right, published by Zoccola et al. 2015) in *S. pistillata*. AEnd, aboral endoderm; CEct, calicoblastic ectoderm; Co, coelenteron; SK, skeleton.

It has been proposed that carbonate is derived from bicarbonate due to a favorable pH in the ECM (Zoccola et al. 2015; McCulloch et al. 2017). Bicarbonate is transported across cell membranes by members of two distinct membrane transporters, the SLC4, and SLC26 transporter families (Zoccola et al. 2015). Previous studies have identified eight potential bicarbonate transporters in corals, of which five (SLC4α–γ) belong to the SLC4 and three to the SLC26 family (the ${\text{SO}}_{4}^{2-}$ transporters, the Cl^−^/${\text{HCO}}_{3}^{-}$ exchangers, and the selective Cl^−^ channels) (supplementary table S1, Supplementary Material online) (Zoccola et al. 2015). A phylogenetic analysis of these genes identified a duplication of the SLC4β gene, termed SLC4γ, in both coral genomes but not in the genomes of the corallimorpharians *A. fenestrafer* and *Discosoma* sp. nor the sea anemones *N. vectensis* and *E. pallida* (fig. 3*a* and *b*). Synteny analysis of all six genomes in our study showed overall high conservation of the genomic locus surrounding SLC4β (fig. 3*b*). Immunolocalization of the ancestral SLC4β gene product revealed ubiquitous expression across all coral tissues, with a somewhat stronger expression in the calicoblastic ectoderm (fig. 3*c*). Immunohistochemical localization of the SLC4γ protein, however, was largely restricted to the calicoblastic ectoderm of the coral *S. pistillata* (fig. 3*c*).

### The Evolution of Carbonic Anhydrases

**Fig. 4.**
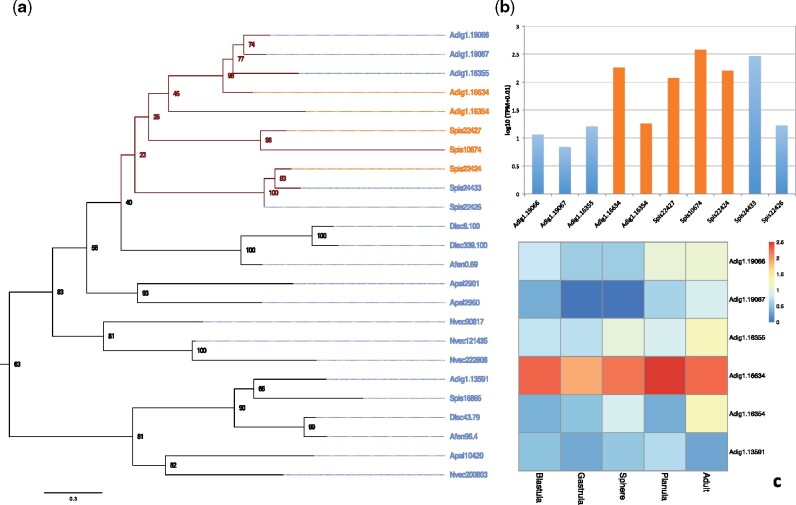
Carbonic anhydrases. (*a*) Phylogeny of CAs across six hexacorallians. Orange labels represent extracellular CAs, and blue labels represent intracellular CAs. (*b*) Expression of CAs across homologs in Scleractinia. Orange labels represent the extracellular CAs, and blue labels represent intracellular CAs. (*c*) Expression of CAs in different developmental stages of *A. digitifera*.

CAs facilitate and catalyze the hydration of metabolic CO_2_ into ${\text{HCO}}_{3}^{-}$ (Moya et al. 2008; Hopkinson et al. 2015). Some coral CAs are secreted directly into the calicoblastic fluid and catalyze this reaction at the site of calcification (Hopkinson et al. 2015). Comparison of the CA repertoire across the six genomes showed frequent lineage-specific expansions, with unique gene duplications being evident across all genomes (fig. 4*c* and supplementary fig. S3 and table S1, Supplementary Material online). However, both scleractinian genomes consistently showed a higher number of duplications of both secreted and membrane-bound CAs (Lin et al. 2017) when compared with corallimorpharians or actiniarians (fig. 4*a*). Lineage-specific duplications of ancestrally cytoplasmic CAs in corals showed newly acquired signatures of extracellular localization echoing the findings of Lin et al. (2017) and suggesting subcellular neolocalization of the respective proteins (fig. 4*a*). Interestingly, we found that extracellular CAs (CA12/CA14) experienced further lineage-specific duplications after the divergence of robust and complex corals (fig. 4*a*). Furthermore, we performed an analysis looking at the expression of these CA homologs across different life stages of the coral *Acropora digitifera* and found that extracellular CAs appear to be generally higher expressed than intracellular CAs in calcifying adult stages (fig. 4*b*) compared with noncalcifying life stages (fig. 4*c*).

### Identification of Core Skeleton Organic Matrix Proteins

**Fig. 5.**
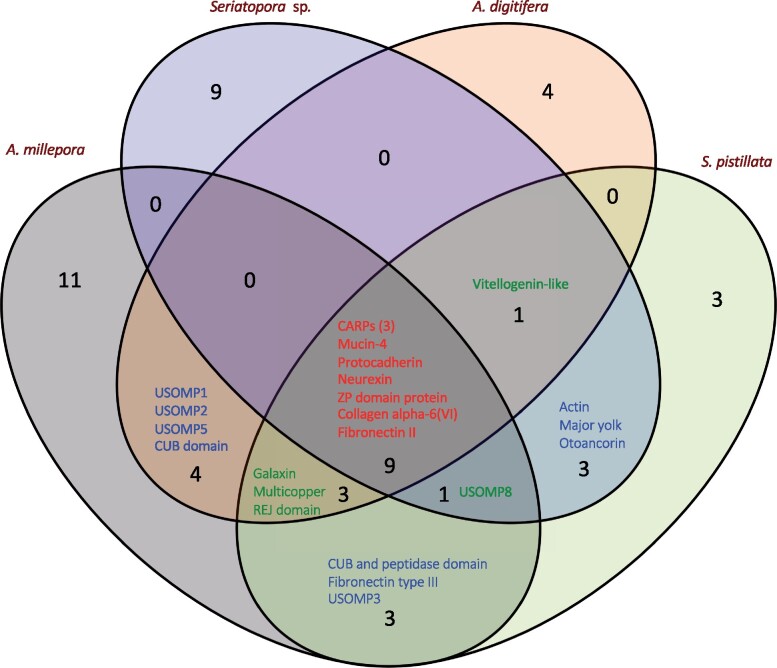
Comparison of the SOMPs identified in four proteomic studies.

To determine a core set of SOMPs that were likely present in the ancestor of reef-building corals, we sequenced the protein constituents of the SOM identified in the skeleton of *S. pistillata* (65 proteins; supplementary table S2, Supplementary Material online) by LC/MS/MS analysis and compared it with proteomic data from three previous studies including Drake et al. (36 SOMPs in *Seriatopora* sp*.* [Drake et al. 2013a, 2013b; Mass et al. 2014; Bhattacharya et al. 2016]), Ramos-Silva et al. (36 SOMPs in *Acropora millepora*), and Takeuchi et al. (30 SOMPs in *Acropora digitifera*). Reciprocal BLASTP analyses between these skeletal proteomes identified nine proteins that were commonly identified in the skeletons of these robust and complex corals (fig. 5 and supplementary table S3, Supplementary Material online). These nine common SOMPs were subsequently defined as the core set of SOMPs that were likely present in the ancestor of both complex and robust corals, whereas all other, noncommon, proteins were defined as species specific SOMPs or potential contamination.

Despite differences in the annotation of these core-SOMPs, most of these genes shared similar domain architectures and, thus, most likely similar functions in Scleractinia. These proteins are three coral acid-rich proteins (CARP4, CARP5, CARP4*γ*), mucin-4 like proteins, protocadherin (protocadherin fat 4), zona pellucida domain-containing protein, neurexin (contactin-associated protein), fibronectin II (MAM and LDL-receptor class A domain–containing protein), and collagen alpha-6 (VI) chain protein (fig. 5). Together, this core set of SOMPs accounted for 67.38% and 69.73% of the total spectral counts in the *S. pistillata* and *A. digitifera* SOM, respectively (supplementary table S4, Supplementary Material online). To further unravel the specific evolutionary mechanisms underlying the recruitment of these proteins to the coral calcification process, we identified and analyzed clear homologous proteins for each of these SOMPs in the genomes of the noncalcifying hexacorallian subclasses Corallimorpharia and Actiniaria.

### Coral Acid-Rich Proteins

**Fig. 6.**
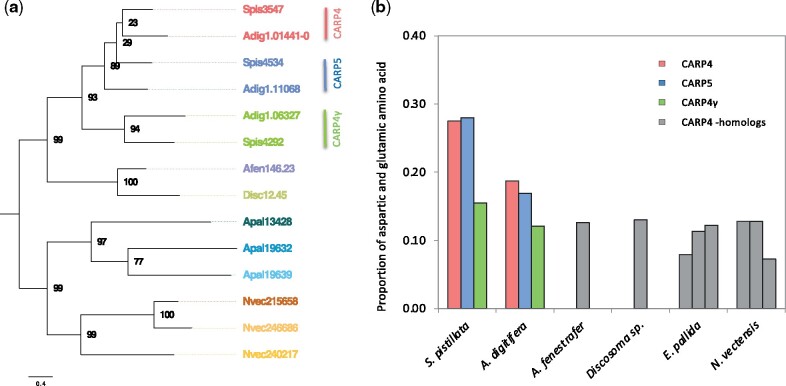
Coral acid-rich proteins (CARPs). (*a*) Phylogenetic analysis of CARP4. (*b*) The abundance of aspartic and glutamic acid residues across the different homologs of CARP4s.

Acidic proteins are critical components for the initiation of calcification, as aspartic acid and glutamic acid have the ability to interact with calcium ions through their negative charge at neutral pH (Addadi and Weiner 1985). All four proteomic data sets consistently identified the coral acid-rich protein 4 (CARP4) as the most abundant protein in the SOM (24% of SOM), whereas the other members of this family, alpha integrin-like protein (CARP4γ, alternatively denoted as CARP4#) and CARP5, showed lower abundance (1.92% and 1.66% of SOM, respectively) (Takeuchi et al. 2016) (supplementary table S4, Supplementary Material online). Previous studies showed that distant homologs of these proteins are found in the sea anemones *Nematostella* and *Anthopleura*, but these studies could not resolve if the observed diversification of these acidic proteins was indeed a coral-specific innovation or already present in the closest noncalcifying relatives (Bhattacharya et al. 2016). Our comparative genomics analysis showed that the last common ancestor of robust and complex corals experienced two scleractinian-specific duplications of the ancestral CARP4 gene after the divergence from Corallimorpharia approximately ∼308 Ma (fig. 6*a*). This gave rise to the three CARP homologs found in the skeletons of contemporary reef-building corals. Interestingly, whereas these gene duplications are specific to corals, and therefore not present in Corallimorpharia nor Actiniaria, we found that both *N. vectensis* and *E. pallida* independently evolved two species-specific duplications (fig. 6*a*). Comparison of the coral-specific duplications to the single homolog present in Corallimorpharia further confirmed that the significant extensions of acid-rich (Asp and Glu [D, E]) amino acids is a specific feature of the coral homologs (Conci et al. 2019). This finding further suggests that these extensions constitute an evolutionary adaption of these novel proteins to their role in the calcification process in Scleractinia (fig. 6*b*). Furthermore, we found that the overall acidity of the coral orthologs generally correlated with their relative abundance in the SOM, that is, the most acidic ortholog CARP4 in *S. pistillata* consistently showed the highest protein abundance in all four studies while the less acidic orthologs, CARP4γ and CARP5, were significantly less abundant (supplementary table S4, Supplementary Material online).

### Transmembrane Proteins

Skeletal transmembrane proteins can facilitate cell–cell and cell–substrate adhesion (Helman et al. 2008). Our broad comparison of secreted transmembrane proteins across the six hexacorallian genomes, such as mucin4 (supplementary fig. S4, Supplementary Material online), procadherin (supplementary fig. S5, Supplementary Material online), neurexin (supplementary fig. S6, Supplementary Material online), and zona pellucida domain proteins (supplementary fig. S7, Supplementary Material online), were consistent in their phylogeny and domain architectures (supplementary table S4, Supplementary Material online). For example, all mucin4-like proteins contained the parallel domain architectures in the C-terminus that are involved in the adherence and anchoring of the cell surface to extracellular matrix (Chaturvedi et al. 2008) (supplementary fig. S4, Supplementary Material online). Protocadherins are composed of two laminin G domains, one cytoplasmic cadherin, and multiple extracellular cadherins (supplementary fig. S5, Supplementary Material online). The two laminin G domains are thought to be involved in cell adhesion (Hohenester et al. 1999), whereas the cadherin domains are involved in Ca^2+^ binding (Takeichi 1988). Another laminin-G domain-containing protein, neurexin, is supposed to connect calicoblastic cells to the extracellular matrix. Neurexin in *S. pistillata* showed similar domain architecture to the actiniarian homologs, whereas the *A. digitifera* ortholog showed higher similarity to the corallimorpharian homologs but appeared to have experienced multiple gene duplications during evolution (supplementary fig. S6, Supplementary Material online). The zona pellucida domain proteins, which are supposed to be responsible for intra- and inter-molecular disulfide bridges and polymerization of proteins, also exhibited highly conserved and identical domain architecture (Boja et al. 2003) (supplementary fig. S7, Supplementary Material online). Despite their conservation in Hexacorallia, these transmembrane proteins still appear to have experienced slight adaptations during or before their co-option to the calcification process. This is evident by the expansion of high repetitive structures and tandem domains, such as thrombospondin type 1 (TSP1) in the N-terminus of mucin4-like proteins (supplementary fig. S4, Supplementary Material online), and laminin G domain in neurexin (supplementary fig. S6, Supplementary Material online).

Among the coral SOMPs, we also identified three conserved proteins that not only have transmembrane domains but also acquired extracellular noncollagenous protein signatures. SOM proteomic data consistently showed high spectral counts for a collagen alpha-6 (VI) (COL6A6) homolog (14.81%), which has been proposed to regulate the epithelial cell-fibronectin interactions (Groulx et al. 2011) and tether aragonite crystals (Nudelman et al. 2010). Hexacorallian phylogenetic analysis of COL6A6 revealed a scleractinian-specific duplication (supplementary fig. S8, supplementary table S4, Supplementary Material online). Protein domain analyses highlighted multiple transmembrane domain insertions and deletions, whereby coral COL6A6 homologues showed different patterns of domain insertions and deletions of cell–cell and cell–substrate adhesion domains such as TSP1 and von Willebrand factor type A (VWA) (supplementary fig. S8, Supplementary Material online). The unique extracellular F5/8 type C (FA58C) domains, inserted in both termini of scleractinian proteins, were found to be the major protein domain in many blood coagulation factors (Groulx et al. 2011). Another fibronectin II domain containing protein was assumed to be the main component of the acid-insoluble and acid-soluble organic matrix of the aragonite skeleton. This protein showed high conservation and similar transmembrane domain architecture to the MAM and LDL-receptor, where these domains exhibited distinctive duplications in corals (supplementary fig. S9, Supplementary Material online). Besides the extension of MAM and LDL-receptor domains, scleractinian homologs also displayed a fibronectin II domain that was completely absent in their corallimorpharian counterparts (supplementary fig. S9, Supplementary Material online).

## Discussion

The fossil record of Scleractinia can be traced back to the early Triassic period around 265 Ma (Ezaki 2000; Simpson et al. 2011; Park et al. 2012). However, our molecular divergence analysis between scleractinians and their closest extant relatives, the Corallimorpharia, suggests that they might have already diverged around 308 Ma (fig. 1*a*–*c*), a finding that is consistent with a common divergence from Actiniaria around 506 Ma but considerably later than suggested in a recent study using Ultra Conserved Elements (Quattrini et al. 2020). Several studies have analyzed the evolutionary origin of Scleractinia using different markers and time calibration methods and the divergence times proposed range from as early >450 Ma (Stolarski et al. 2011; Huang and Roy 2013) to more recent estimates <283 Ma (Hedges et al. 2006; Simpson et al. 2011; Park et al. 2012), placing our divergence estimates somewhere in between. However, it is important to note that there’s currently no fossil evidence to support the presence of calcifying scleractinian like corals before ∼265 Ma (Ezaki 2000). Based on our analyses, we therefore conclude that Scleractinia likely evolved the ability calcify some time during the ∼43 My between the divergence from the noncalcifying Corallimorpharia (308.37 Ma) and the earliest evidence of scleractinian like corals in the fossil record 265 Ma (Ezaki 2000). Furthermore, it has previously been proposed that the evolution of coral calcification might have been driven by the co-option of existing genes, which is strongly supported by our study. Specifically, our analyses show that the currently known proteins involved in coral calcification were either already present in the noncalcifying ancestor of corals or evolved through coral-specific gene duplication events.

### The Co-option of Genes as Evolutionary Mechanism of Coral Calcification

The continuous supply of calcium and carbonate ions to the ECM is a critical factor for the growth of coral skeletons (McCulloch et al. 2017; Drake et al. 2020). This process is controlled by Ca^2+^-ATPase pumps, bicarbonate anion transporters (Furla et al. 2000; Zoccola et al. 2015), and CAs (Moya et al. 2008; Bertucci et al. 2011; 2013; Zoccola et al. 2015) for which corals have evolved specific adaptations. These adaptations included the co-option of existing genes, such as the Ca^2+^ transporting calcium ATPases PMCA1-3, as well as the neo-functionalization of coral-specific gene duplications, such as the coral-specific bicarbonate transporter SLC4γ. This is strongly supported by the calicoblastic ectoderm-specific expression of the respective coral homolog that suggests that this protein has been recruited to the calcification process to facilitate the transport of calcium and bicarbonate to the site of calcification.

Although efficient transport of Ca^2+^ and CaCO_3_ maintains a high saturation of ions in the ECM, the SOM secreted by the calicoblastic cells also contains key proteins to stabilize ACC. These proteins can support nucleation and growth/inhibition of crystals to provide a connection between the calicoblastic cells and the skeleton, thereby directing skeleton growth and morphological differentiation (Falini et al. 2015). The high diversity of SOMPs identified across the different coral species could, therefore, suggest that some of the proteins found only in one, or some of the coral skeletons might contribute to more specific functions such as determining or contributing to the distinct coral morphologies (Ramos-Silva et al. 2013; Drake et al. 2013a). However, it should be noted that while they persist in skeletal tissues that have been thoroughly cleaned with sodium hypochlorite before extraction, the generally low abundance of these species-specific SOMPs might just as well point toward potential contaminants. The conserved SOMPs between robust corals and complex corals identified in this study, however, provided the opportunity to trace the evolutionary origin and history of these common coral calcification genes. The fact that these coral-specific homologs also constitute the most abundant proteins in the SOM of both robust and complex corals is a strong indicator for their essential role in the calcification process (supplementary table S4, Supplementary Material online and fig. 5).

Coral acid-rich proteins are the most abundant SOMPs (Mass et al. 2013; Von Euw et al. 2017) and show scleractinian-specific duplications and subsequent evolutionary adaptations. These duplicated acidic proteins have further undergone extensive divergence in the scleractinian lineage through the expansion of aspartic and glutamic acid stretches, leading to unique species-specific sets of acidic paralogs. Their ability to catalyze the precipitation and stabilization of ACC in vitro, as well as the presence of distinct CARPs during the different stages of mineral formation (Mass et al. 2013; Kocot et al. 2016; Von Euw et al. 2017), makes it plausible to assume that they constitute one, if not the, central evolutionary innovation that enabled the ancestor of contemporary scleractinian corals to calcify.

Our data further revealed that scleractinian calcification only required the co-option of a small number of transmembrane proteins, including mucin4, procadherin, neurexin, and zona pellucida domain protein. These transmembrane proteins can perform adhering functions between calicoblastic cells, newly formed skeletons, and skeleton-blanket matrix (Helman et al. 2008; Ramos-Silva et al. 2013; Falini et al. 2015; Takeuchi et al. 2016). Their high conservation further indicates that these transmembrane proteins did not experience gene duplications but rather small adjustments of their encoded domains. We also observed a small amount of extracellular matrix proteins in the SOM. These proteins were also present in the noncalcifying hexacorallians and, based on their conserved domain composition, they all appear to have retained their ancestral function. In contrast, we find that some extracellular matrix proteins, such as COL6A6 and fibronectin II domain-containing proteins, appeared to have acquired novel domain insertions, and subsequent gene duplications, likely as adaptation to species-specific traits.

### Domain Duplications Are Important for the Evolution of Coral Calcification

Previous studies suggested that domain shuffling might have been a general evolutionary mechanism underlying the evolution of coral calcification (Ramos-Silva et al. 2013; Takeuchi et al. 2016). Our analysis of SOMPs highlights that most of these genes share a conserved domain composition with their homologs in soft-bodied relatives and were likely co-opted to the processes of coral calcification. However, a comparative analysis of critical SOMP domains (supplementary table S5, Supplementary Material online) showed that the overall number of these domains is significantly higher in coral homologs compared with corallimorpharians, suggesting that domain duplications might indeed have contributed to the evolution of coral calcification. Furthermore, the striking preponderance of highly repetitive, and low complexity transmembrane domains, such as TSP, MAM, LDL-receptor, or EGF-like domains, is reminiscent of the rapid evolving secretomes of sea shells (Kocot et al. 2016). Taken together, our findings confirm that domain duplications were likely important for the evolution of coral calcification.

### The Evolution of Calcification in Corals and the Ancestral Biomineralization Toolkit

Our evolutionary analysis of coral calcification genes shows that the basic functions required to precipitate calcium carbonate and coordinate its deposition were already encoded in the genome of the soft bodied ancestor of hexacorallians more than 500 Ma. Many of the proteins involved in the calcification process, as identified here, are in fact members of ancient gene families that provide essential functions required for the general functioning of eukaryotic cells (Knoll 2003). This echoes the findings in other calcifying animal lineages from the early Cambrian, a time when calcified skeletons appeared independently across diverse animal clades (Knoll 2003; Murdock and Donoghue 2011; Murdock 2020). As such, our findings support the hypothesis of an “ancestral biomineralization toolkit” (Murdock 2020) comprised of basic eukaryotic gene functions for the continuous provision of ions to the calcification process and SOMP, such as acidic proteins, to initiate and control the precipitation process. This basic toolkit allowed a variety of animal lineages to evolve calcium carbonate skeletons independently throughout evolution and was likely a major driver of metazoan diversification (Smith and Harper 2013).

## Conclusion

The evolution of calcification was an essential innovation that transformed the soft-bodied ancestors of corals into the important ecosystem builders they are today. Our comparative genomic analyses of contemporary corals and their closest noncalcifying relatives show that this transformative innovation did likely not require the evolution of extensive genetic novelties but rather highlights the role of lineage specific gene duplications and the co-option of existing genes to the process. The requirement of comparably few genetic adaptations provides an explanation for the ubiquity of calcification within Cnidaria as well as across the animal kingdom.

## Materials and Methods

### Data Collection, Gene Family Analysis, and Divergence Time Estimation

The complete cnidarian genomes and gene models of *A. digitifera* (PRJNA314803, PRJDA67425), *S. pistillata* (PRJNA415215*)*, *A. fenestrafer* (PRJNA354436), *Discosoma* sp. (PRJNA354492), *N. vectensis* (PRJNA19965, PRJNA12581), *E. pallida* (PRJNA386175), and *H. magnipapillata* (PRJNA12876) were collected on NCBI and are available at http://reefgenomics.org (last accessed May 24, 2021) (Liew et al. 2016). Three complete Symbiodiniaceae genomes and gene models of *Breviolum minutum* (PRJDB732), *Symbiodinium microadriaticum* (PRJNA292355), and *Fugacium kawagutii* (PRJNA556154) were also collected on NCBI. In order to prepare the gene models for the subsequent comparative analyses, we discarded alternative isoforms and only selected the longest one for each gene. Further, we removed proteins with fewer than ten amino acids or low sequence quality (more than 20% stop codons, more than 20% nonstandard amino acids).

After preparing the final gene and corresponding protein sets, OrthoMCL (Li et al. 2003) was run using an *e*-value cut-off of 10^−5^ to create groups of orthologs and paralogs across all seven genomes (*H. magnipapillata* included as outgroup) that were subsequently assigned to the latest OrthoMCL-DB v4 (Chen et al. 2006) for further validation. Protein sequences from final orthologous groups were aligned with MUSCLE v3.8.31 (Edgar 2004) using default settings and trimmed using TrimAl v1.4 with automated1 (Capella-Gutiérrez et al. 2009). To build the phylogenetic trees, we determined the best model with Prottest v3.4 (Darriba et al. 2011) and performed phylogenetic analysis using RAxML v8.1.22 (Stamatakis 2014) with 1,000 bootstraps. The final trees were visualized and modified using FigTree v1.4.3.

Corresponding coding and amino acid sequences were retrieved from orthologs mentioned above. Multiple protein sequences within each group were aligned using MUSCLE with default parameters. Nucleotide alignments were generated from the alignment of corresponding proteins using customized ParaAT scripts (Zhang et al. 2012). Then synonymous substitutions were calculated using the codeml program from PAML v4.8 (Yang 2007). To estimate the divergence, the *Ks* distributions of pair-wise orthologs were finally visualized by ggplot2 (Wickham 2016). To estimate the age of the ancestral divergence and the rate of evolution on each lineage, we mainly applied BEAST v2.6.0 (Bouckaert et al. 2014) for Bayesian evolutionary analysis. bModelTest (Bouckaert and Drummond 2017) were used to select the most appropriate substitution model. Calibrations of a set of taxa were specified according to the fossil record from Timetree (Ezaki 2000; Hedges and Kumar 2009; Miglietta et al. 2010; Park et al. 2012) (http://www.timetree.org, last accessed May 24, 2021). The final trees were visualized, analyzed, and modified using Tracer v1.7.1 and FigTree v1.4.3.

### Transcriptome Analysis

To check the absence or presence of our candidate homologs, we further quantified the RNA-seq expression across six hexacorallian genomes. Raw RNA-seq reads from adult stages of six hexacorallians, including *A. digitifera* (PRJDB3244) (Reyes-Bermudez et al. 2016), *S. pistillata* (PRJNA415215) (Voolstra et al. 2017), *A. fenestrafer* (PRJNA354436) (Wang et al. 2017), *Discosoma* sp. (PRJNA354492) (Wang et al. 2017), *N. vectensis* (PRJNA189768) (Helm et al. 2013), and *E. pallida* (PRJNA386175) (Baumgarten et al. 2015), were downloaded from NCBI. The complete genomes and gene models of *A. digitifera*, *S. pistillata*, *A. fenestrafer*, *Discosoma* sp., *N. vectensis*, and *E. pallida* were also downloaded accordingly. To better understand the changes in certain gene expression affecting calcification stages, we also collected published raw data in embryonic, larval and adult samples to characterize stage-specific transcription profiles. Those samples included Blastula (irregular cellular bilayer), Gastrula (germ layer formation), Sphere (initiation larval life and establishment of cellular lineage), Planula (cell diversification), and calcifying adult (calcifying and diversity of cell populations) in *A. digitifera* (PRJDB3244) (Reyes-Bermudez et al. 2016).

To avoid bias that resulted from disparate bioinformatics tools in quantifying gene expression, sequence raw reads from different data sets were processed with identical analytical pipelines. Briefly, raw RNA-seq reads were trimmed using Trimmomatic v0.32 (Bolger et al. 2014) and quality-checked using FastQC v0.11.3 (Andrews 2010). Two different methods were applied to assemble and quantify the filtered RNA-seq reads. To acquire a complete transcriptome model, we applied an RNA-seq de novo assembly using Trinity. However, to accurately check the existence of the genes and quantify their expression, we utilized a reference-based strategy. Briefly, gene expression levels (TPM, transcripts per million) were quantified through alignments to their corresponding gene models using Kallisto v0.42.2 (Bray et al. 2016). Any orthologous genes showing duplication/elimination events were further validated based on their expression. We further confirmed ambiguous genes that had significant different domain architectures across six genomes using the assembled transcriptome model. To further enable direct comparison of gene expression values in different developmental stages in *A. digitifera*, differential gene expression analysis was inferred from the mapping counts using the edgeR R package. TPM values were also normalized with the median ratio across different developmental stage samples and species samples. The heat map and clustered matrix were created using R with Bioconductor and pheatmap (Kolde 2012).

### Proteomic Data Sets to Define Conserved SOMPs in Corals

Organic matrix proteins were extracted as published previously (Tambutté et al. 2015). Coral branches were treated with sodium hypochlorite to prepare skeletons then cryo-ground into powder. The powder was incubated with sodium hypochlorite to remove potential contaminants such as endoliths. Thereafter, the powder was demineralized in EDTA and the obtained solution was filtered through Sep-Pak Plus C18 cartridges (Waters, 5 kDa). Protein content was determined using the bicinchoninic acid assay kit (BC Protein Assay, Interchim). A standard curve was established with bovine serum albumin and the absorbance was measured with a microplate reader (Epoch, BioTec, US) at 562 nm. Extracted protein fractions were run on a SDS-page and submitted to the KAUST Proteomic core lab for proteomic analysis. The proteins were in gel Trypsin digested overnight and the peptides were finally resuspended in 20 μl of sample buffer (3% ACN, 0.1% formic acid).

The NanoLC MS/MS analysis was performed on an online system consisting of a nano-pump UltiMate 3000 UHPLC binary HPLC system (Dionex, ThermoFisher) coupled to a Q-Exactive HF mass spectrometer (ThermoFisher, Germany). A total of 2 μl of the peptide per sample was injected into a precolumn 300 µm × 5 mm (Acclaim PepMap, 5 µm particle size). After loading, peptides were eluted to an Acclaim PepMap100 C18 capillary column (75 µm × 15 cm, 100 Å, 3 μm particle sizes). Peptides were eluted into the MS, at a flow rate of 300 nl/min, using a 40-min gradient from 5% to 40% mobile phase B. Mobile phase A was 0.1% formic acid in H_2_O and mobile phase B was 80% acetonitrile and 0.1% formic acid. The mass spectrometer was operated in positive and data-dependent mode, with a single MS scan (350–1,400 *m*/*z* at 60,000 resolution [at 200 *m*/*z*] in a profile mode) followed by MS/MS scans on the ten most intense ions at 15,000 resolution. Ions selected for MS/MS scan were fragmented using higher energy collision dissociation at normalized collision energy of 28% and using an isolation window of 1.8 *m*/*z*.

### Protein Identification

The RAW files from Q-Exactive HF were converted into Mascot generic format (mgf) files using Proteome Discoverer version 1.4 (Thermo Scientific). These files were submitted to MASCOT v2.3 (Matrix Sciences Ltd, United Kingdom) for database search against a *S. pistillata* genome database based on the predicted genes in the published *S. pistillata* genome (Liew et al. 2016; Voolstra et al. 2017). The mass tolerance was set to 20 ppm for precursors, and 0.5 Da for the MS/MS fragment ion. The fixed modifications were set to carbamidomethyl and variable modifications were set to oxidation at methionine. The MASCOT result files were processed using Scaffold v4.1.1 (Proteome Software Inc. USA) software for validation of peptide and protein identifications with a threshold of 95% using the Prophet algorithm. This approach detected 65 SOMPs (supplementary table S2, Supplementary Material online), of which 48 had strong support (spectrum counts in at least two of four samples).

Homology analyses were performed using the identified *S. pistillata* proteomic sequence against integrated proteomic data from three previous studies including Drake et al. (36 in *Seriatopora* sp. [Bhattacharya et al. 2016], formerly denoted *S. pistillata* in Drake et al.), Ramos-Silva et al. (36 in *A. millepora*), and Takeuchi et al. (30 in *A. digitifera*). We performed BLASTP searches using default parameters and OrthoMCL pipeline to determine orthologous/paralogous gene families across these proteomic studies. Using this approach, we identified nine core SOMPs that were commonly identified in the skeletons of all four coral species.

### The Evolution of Calcification Genes

Based on previously published data, we established a list of putative ion transporters involved in calcium and bicarbonate supply for coral calcification based on the analogies with transporters previously described in mammals, including calcium-transporting ATPase (ATP2B, ATP2C), Inositol 1,4,5-trisphosphate receptor type (ITPR1), stromal interaction molecule (STIM1), calcium ion channel family (ORAI, CACNA2D, CAC), sodium/calcium exchanger (SLC8A), bicarbonate transporter (SLC4), CA, and Calreticulin (supplementary table S1, Supplementary Material online). The core set of conserved SOMPs was identified in four proteomic data sets described above (supplementary table S4, Supplementary Material online).

Homology analysis of ion transporters and SOMPs was performed with local BLASTP searches against the predicted coding genes of *A. digitifera*, *S. pistillata*, *A. fenestrafer*, *Discosoma* sp., *N. vectensis*, and *E. pallida* (*e*-value <10^−5^). The best matches of each SOMP and ion transporter were also manually compared on the level of domain architecture and genomic synteny. Corresponding sequences from each species were selected from the BLASTP searches against the known candidates and further validated against the ortholog/paralogous groups. To further validate their existence, we checked the expression levels (log_10_(TPM + 0.001)) across all homologous genes based on the transcriptome data described above. To understand how the changes in those ion transporters and SOMPs regulated morphogenetic transitions (especially calcifying), we also characterized stage-specific transcription profiles using normalized TPMs at different developmental stages of *A. digitifera.* Using those homologous genes, phylogenetic analyses of those proteins were reconstructed following the pipeline describe above. To obtain the domain annotations, we used InterProScan (Jones et al. 2014) against various databases, including Pfam, ProDom, PRINTS, and SMART. Gene Ontology was obtained from the BLASTP results. We also used Phobius (Jones et al. 2014) to determine the location of each gene. Additional functional information of pathways were derived from Kyoto Encyclopaedia of Genes and Genomes (Kanehisa 2002). For potential interesting genes, phylogenetic trees were built using the same method described above.

Due to the inaccuracy of some gene models, we also selected the proteins in the trees that disagreed with the expected phylogeny or domain architectures. We then searched orthologous protein against the de novo transcriptomes or further corresponding genomes using TBLASTN. We manually supplemented and modified some proteins, such as extracellular CA in *Discosoma* sp. and *A. fenestrafer*, acidic proteins in *A. digitifera*, mucin4-like in *S. pistillata*, Procadherin in *A. digitifera*, collagen alpha-6 (VI) in *A. digitifera*, and all fibronectin II proteins.

### Immunolocalization

Polyclonal antibodies against PMCA1-3 were produced in rabbit by Eurogentec. Antibodies were raised against the following peptides: CLTGESDLVKKGPDRD and CLIRDSSGKVSQKKFD for PMCA1, CREKFGKNFMPLEPPR and CDRLMNYKP YGRHKPL for PMCA2, and CYKKQEGKPKDSGQGF and CTVTPAAEEYSMTTGN for PMCA3. Apexes of colonies were prepared for immunolocalization as described previously (Moya et al. 2008; Bertucci et al. 2011). Species were fixed in 3% paraformaldehyde in S22 buffer at 4 °C overnight and then decalcified using EDTA in Ca-free S22 at 4 °C. They were then dehydrated in an ethanol series and embedded in Paraplast. Cross-sections (6 µm thick) were cut and mounted on silane-coated glass slides. Then, deparaffinized sections of tissues were incubated for 1 h in blocking medium (1% BSA, 0.2% teleostean gelatin, 0.05% Tween 20 in phosphate-buffered saline [PBS] pH 7.4) at RT. The samples were then incubated with the anti-PMCAs or the preimmune serum as primary antibodies. After rinsing in in blocking medium, samples were incubated with biotinylated antirabbit antibodies as secondary antibodies. All samples were subsequently stained with streptavidin-Alexa Fluor 568 (Molecular Probes, Invitrogen), and DAPI 0.002% (4′6-diamidino-2-phenylindole, Sigma) was used to stain the nuclei. Samples were embedded in Pro-Long antifade solution (Molecular Probes, Invitrogen) and analyzed with a confocal laser scanning microscope (Leica SP5) equipped with UV and visible laser lines.

## Supplementary Material

Supplementary data are available at *Molecular Biology and Evolution* online.

## Supplementary Material

msab103_Supplementary_Data
