# Supplementary material for: The Evolution of Calcification in Reef-Building Corals
Source: Mol Biol Evol. 2021 Apr 19;38(9):3543–55. doi: 10.1093/molbev/msab103 (PMC8382919; doi:10.1093/molbev/msab103)
Supplement: msab103_Supplementary_Data [file msab103_supplementary_data.zip › Supplementary Figures.pdf]

Supplementary Figures

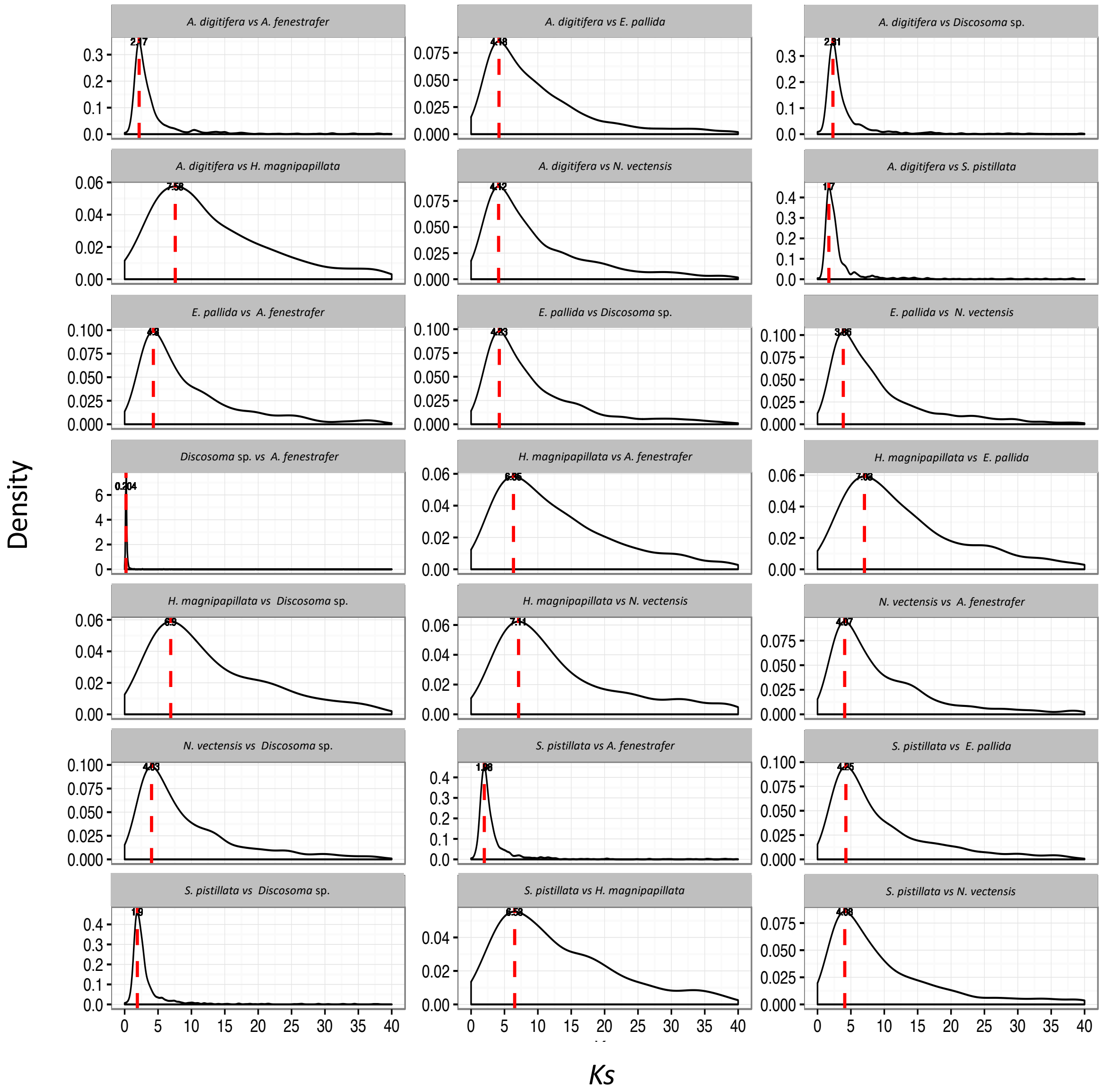

Fig. S1 Density of pair-wise genetic divergence calculated based on synonymous substitution rates (*Ks*) of 1421 orthologous genes across six hexcorallian genomes. *H. magnipapillata* placed as out group.

ScholarOne, 375 Greenbrier Drive, Charlottesville, VA, 22901 Support: (434) 964-4100

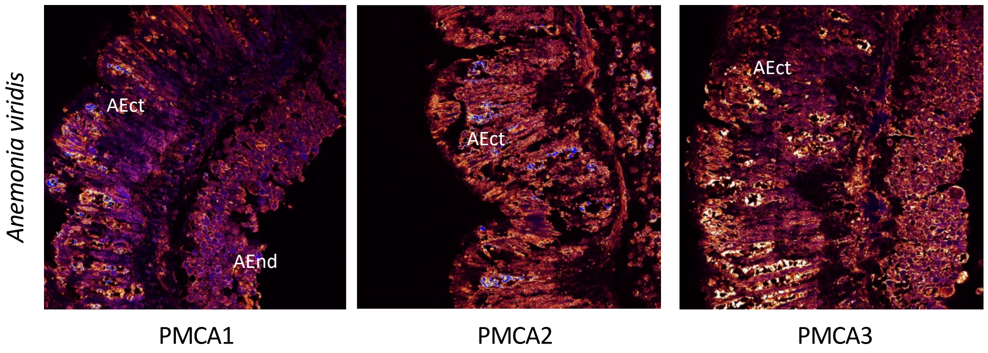

Fig. S2 Immunolocalization of PMCA1, PMCA2 and PMCA3 in the sea anemone *Anemonia viridis*. AEct = Aboral Ectoderm; AEnd = Aboral Endoderm.

Proof: Mol. Biol. Evol.

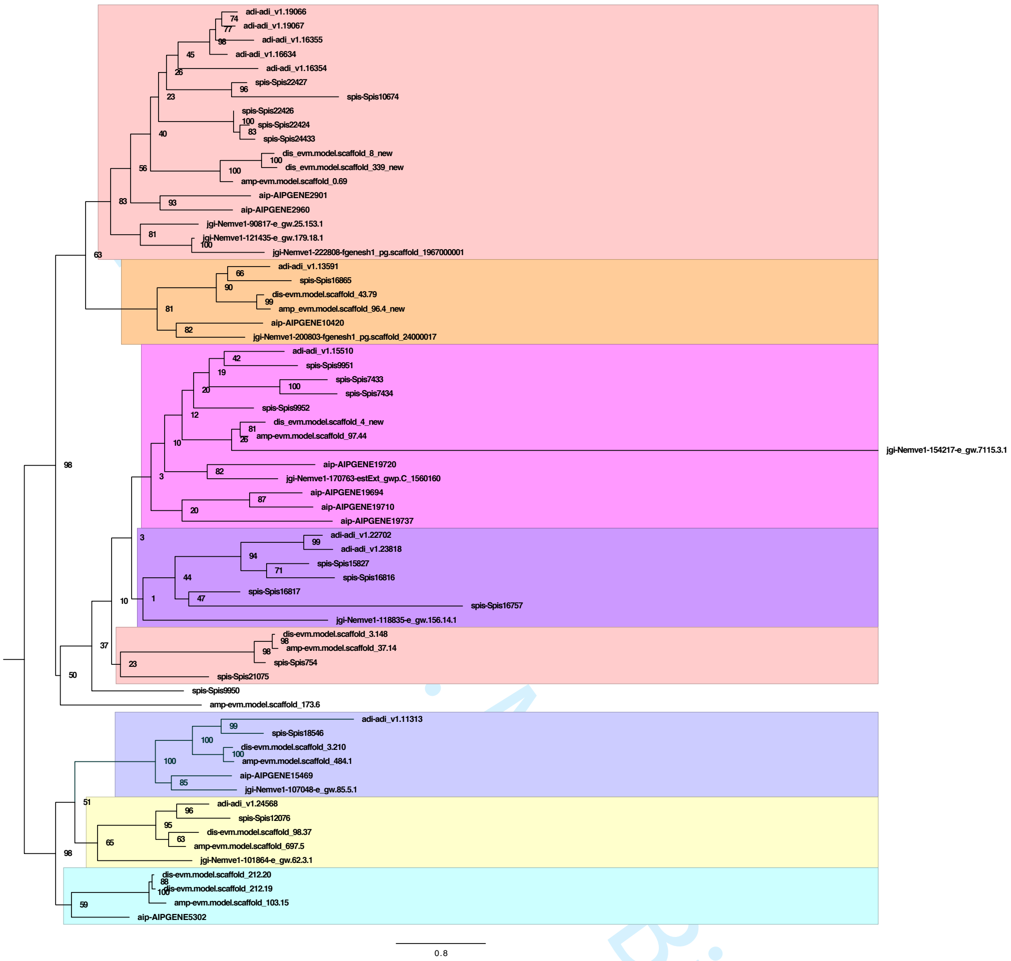

Fig. S3 Phylogenetic tree of carbonic anhydrases across six hexacorallian genomes.

ScholarOne, 375 Greenbrier Drive, Charlottesville, VA, 22901 Support: (434) 964-4100

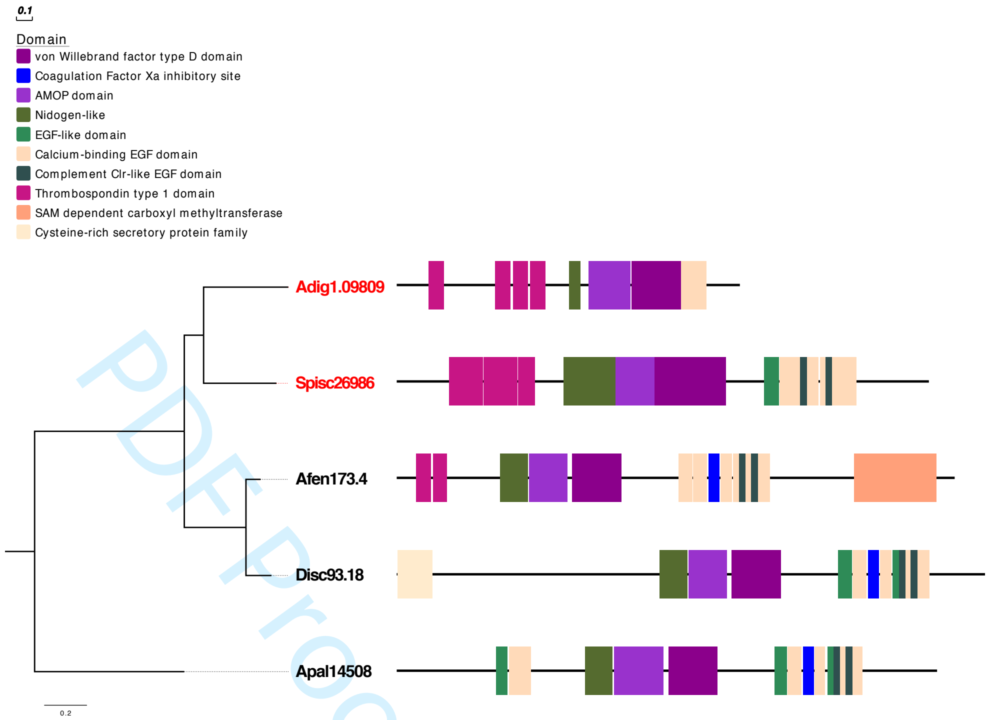

Fig. S4 Phylogenetic analysis and domain distribution of Mucin4-like protein. Red labels indicate their existence across four SOMPs (skeleton organic matrix proteins) studies. These four SOMPs datasets, integrating our *S. pistillata* proteomic sequence and 3 previous studies including Drake et al. (36 in *Seriatopora* sp. (Bhattacharya, et al. 2016), formerly denoted *S. pistillata* in Drake et al.), Ramos-Silva et al. (36 in *A. millepora*) and Takeuchi et al. (30 in *A. digitifera*).

PDF Proof: Mol. Biol. Evol.

ScholarOne, 375 Greenbrier Drive, Charlottesville, VA, 22901 Support: (434) 964-4100

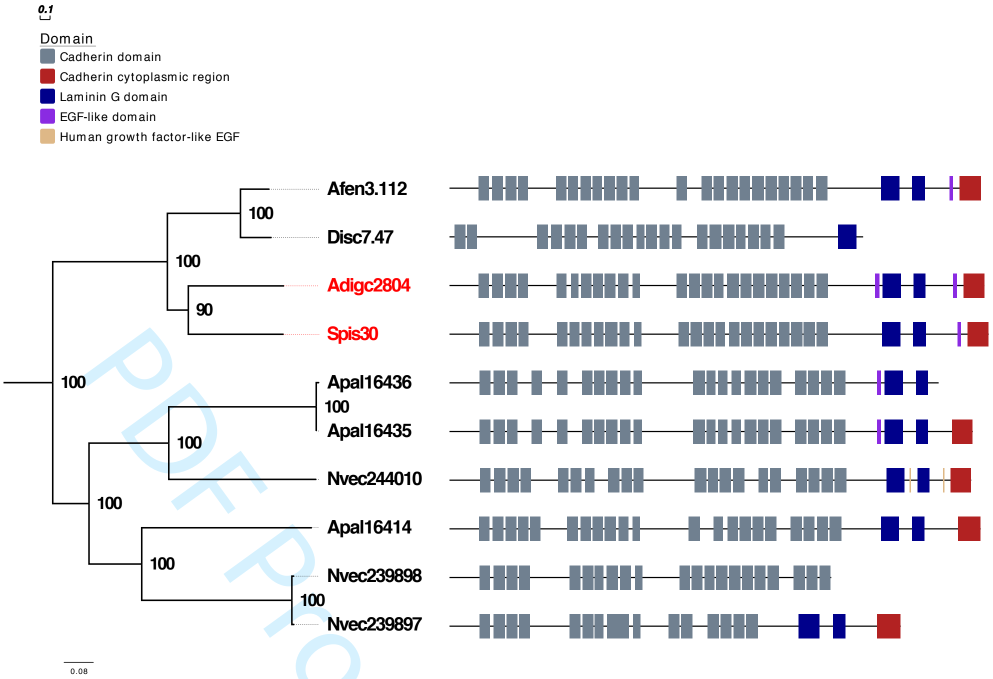

925

926 **Fig. S5** Phylogenetic analysis and domain distribution for Protocadherin orthologous.

927 Red labels indicate their existence across four SOMPs experiments.

PDF Proof. Mol. Biol. Evol.

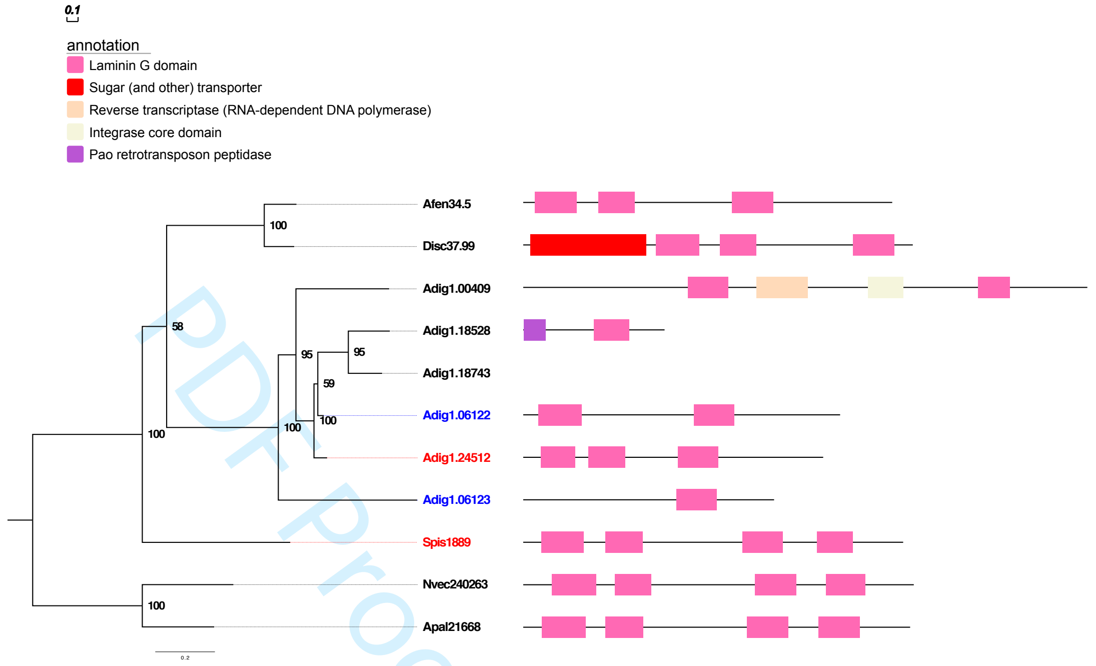

Fig. S6 Phylogenetic analysis and domain distribution of contactin-associated protein (Neurexin). (Red one indicates those genes showed up in all four SOM proteomic datasets, blue is in some of them).

PDF Proof: Mol. Biol. Evol.

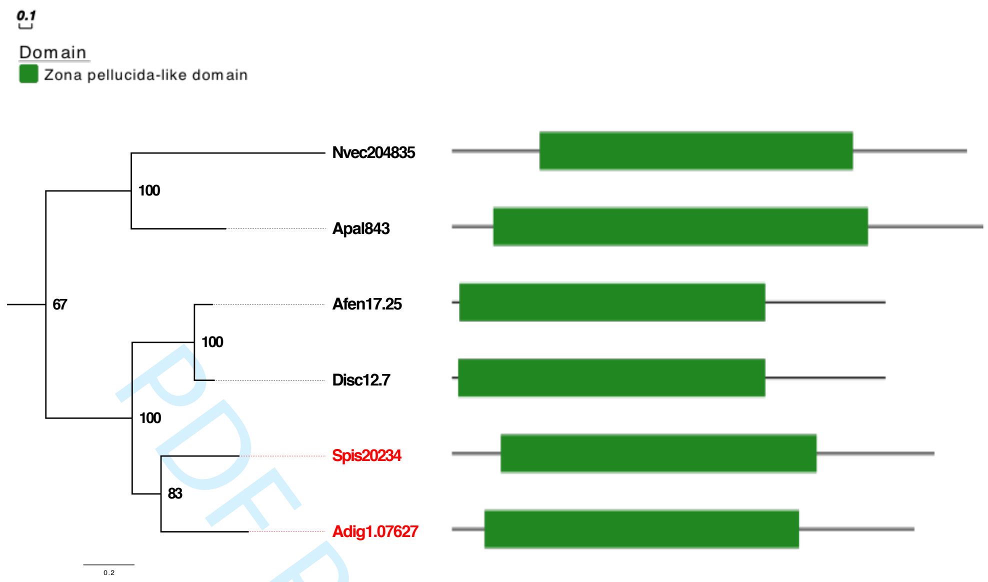

933

934

935 **Fig. S7** Phylogenetic analysis and domain distribution of Zona pellucida domain protein.

936 Red labels indicate their existence across four coral SOMPs studies.

PDF Proof: Mol. Biol. Evol.

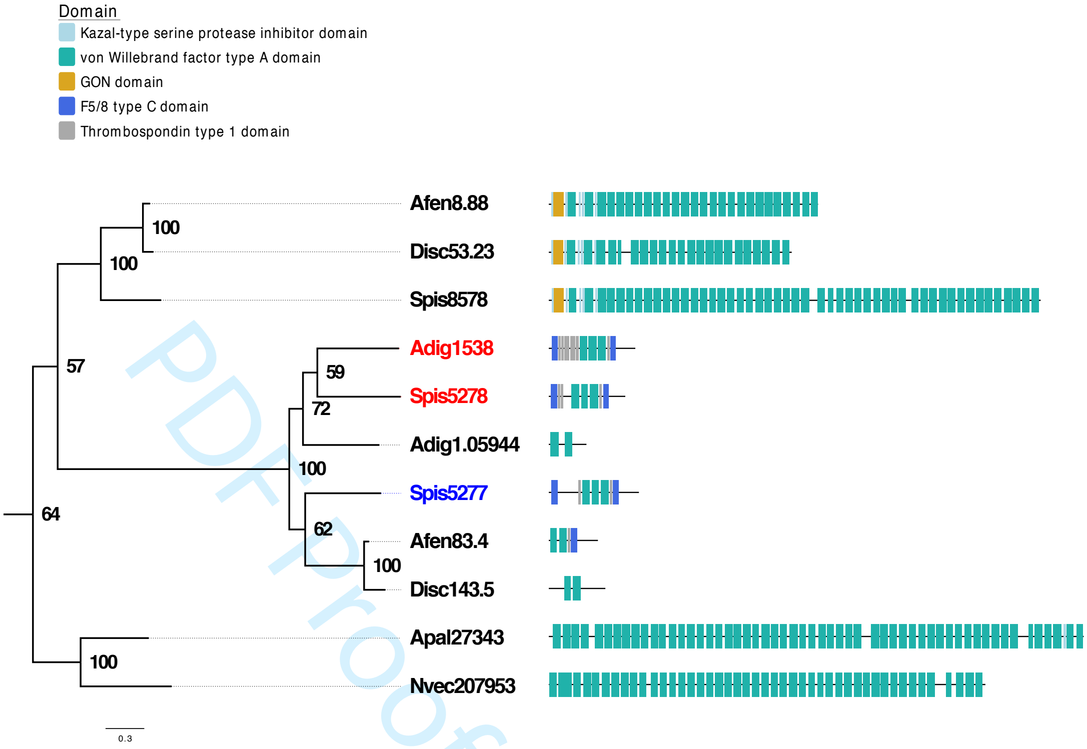

937

938

939

940 **Fig. S8** Phylogenetic analysis and domain distribution of collagen alpha-6 (VI). Red labels indicate their existence across four SOMPs experiments.

PDF Proof: Mol. Biol. Evol.

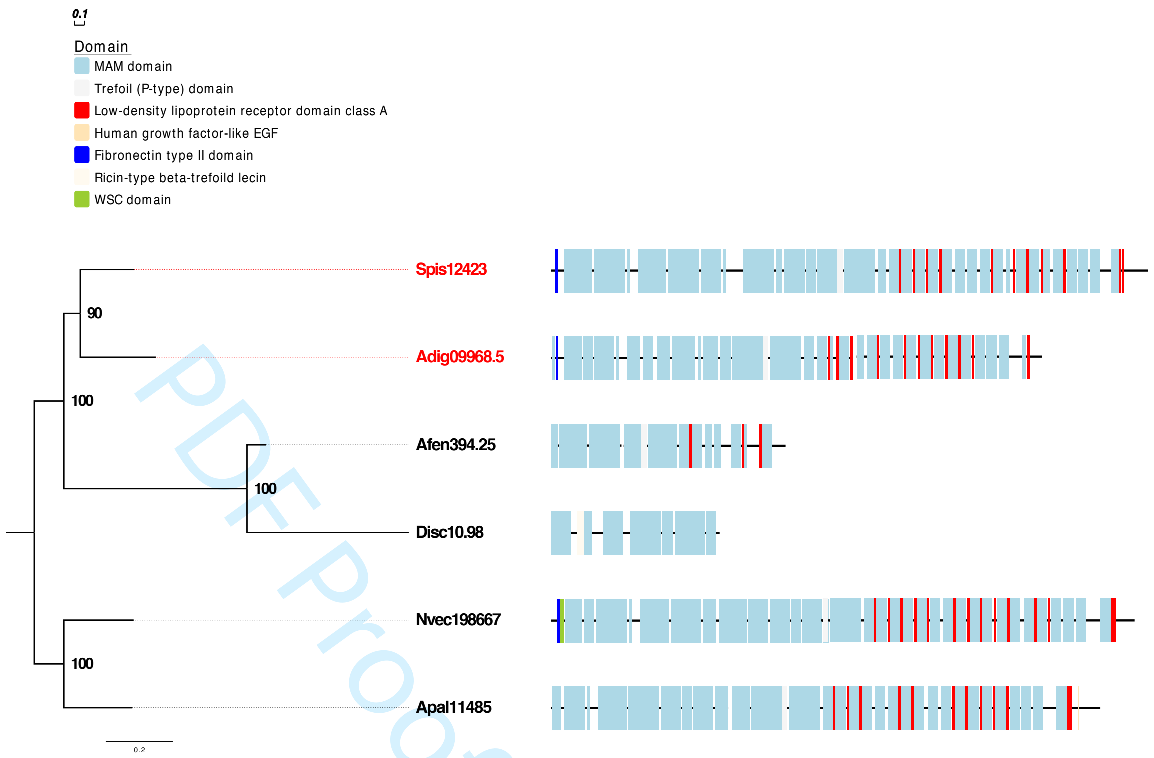

Fig. S9 Phylogenetic analysis of fibronectin II (MAM and LDL-receptor class A domain-containing protein) and corresponding protein domain distribution. Red labels indicate their presence across four SOMPs experiments.

PDF Proof Mol. Biol. Evol.
